# Building a Shared, Scalable, and Sustainable Source for the Problem-Oriented Medical Record: Developmental Study

**DOI:** 10.2196/29174

**Published:** 2021-10-13

**Authors:** Christophe Gaudet-Blavignac, Andrea Rudaz, Christian Lovis

**Affiliations:** 1 Division of Medical Information Sciences Geneva University Hospitals Geneva Switzerland; 2 Department of Radiology and Medical Informatics University of Geneva Geneva Switzerland; 3 Medical and Quality Directorate Geneva University Hospitals Geneva Switzerland

**Keywords:** medical records, problem-oriented, electronic health records, semantics

## Abstract

**Background:**

Since the creation of the problem-oriented medical record, the building of problem lists has been the focus of many studies. To date, this issue is not well resolved, and building an appropriate contextualized problem list is still a challenge.

**Objective:**

This paper aims to present the process of building a shared multipurpose common problem list at the Geneva University Hospitals. This list aims to bridge the gap between clinicians’ language expressed in free text and secondary uses requiring structured information.

**Methods:**

We focused on the needs of clinicians by building a list of uniquely identified expressions to support their daily activities. In the second stage, these expressions were connected to additional information to build a complex graph of information. A list of 45,946 expressions manually extracted from clinical documents was manually curated and encoded in multiple semantic dimensions, such as International Classification of Diseases, 10th revision; International Classification of Primary Care 2nd edition; Systematized Nomenclature of Medicine Clinical Terms; or dimensions dictated by specific usages, such as identifying expressions specific to a domain, a gender, or an intervention. The list was progressively deployed for clinicians with an iterative process of quality control, maintenance, and improvements, including the addition of new expressions or dimensions for specific needs. The problem management of the electronic health record allowed the measurement and correction of encoding based on real-world use.

**Results:**

The list was deployed in production in January 2017 and was regularly updated and deployed in new divisions of the hospital. Over 4 years, 684,102 problems were created using the list. The proportion of free-text entries decreased progressively from 37.47% (8321/22,206) in December 2017 to 18.38% (4547/24,738) in December 2020. In the last version of the list, over 14 dimensions were mapped to expressions, among which 5 were international classifications and 8 were other classifications for specific uses. The list became a central axis in the electronic health record, being used for many different purposes linked to care, such as surgical planning or emergency wards, or in research, for various predictions using machine learning techniques.

**Conclusions:**

This study breaks with common approaches primarily by focusing on real clinicians’ language when expressing patients’ problems and secondarily by mapping whatever is required, including controlled vocabularies to answer specific needs. This approach improves the quality of the expression of patients’ problems while allowing the building of as many structured dimensions as needed to convey semantics according to specific contexts. The method is shown to be scalable, sustainable, and efficient at hiding the complexity of semantics or the burden of constraint-structured problem list entry for clinicians. Ongoing work is analyzing the impact of this approach on how clinicians express patients’ problems.

## Introduction

### Background

The concept of a problem-oriented medical record is as old as 1968 [1]. One of the key elements of this approach is a list of relevant problems, current or past, which are important for understanding the patient’s condition. A problem can be anything, complaints, symptoms, existing or previous conditions, diagnosis, procedures, socioeconomic issues, etc. This list is the corner stone of the clinician’s education and the patient record. It is used from the first encounter, where it is named *chief complaint* to drive clinical reasoning but increasingly to support electronic decision support and diagnostic or care pathways. With the widespread adoption of electronic health records (EHRs) and since the Meaningful Use Act established the problem list as a requirement for care facilities [2,3], it has been the focus of much research and multiple improvements. However, its digitization has brought new opportunities and challenges. Problem lists vary in time and are influenced by the conditions of the population a care facility deserves or the specialties covered.

### Creation and Evolution

The building of a problem list can be driven by free-text entries made by clinicians or by the creation of a finite list of items from which they can choose. Terms included in these premade lists are often taken from existing terminology. Compared with the use of free text, a premade list allows for more structured data and easier secondary use [4-6]. The use of the Systematized Nomenclature of Medicine Clinical Terms (SNOMED CT) [7,8] for the problem list seems to provide good coverage [9-11]. In 2009, the Clinical Observations Recording and Encoding Problem List Subset of SNOMED CT was created using data from 8 institutions [12]. The terms extracted from those institutions were mapped to the SNOMED CT concepts to create a subset usable as a problem list. The current version contains 6565 SNOMED CT concepts. Other approaches have been explored, such as the automatic generation of a patient’s problem list using natural language processing and international terminologies but with lists of less than 200 problems focused on a clinical specialty [13-17]. When creating a problem list, the equilibrium between a list representing what care professionals need to express and an interoperable controlled vocabulary is difficult to find [18].

Using terminology such as the International Classification of Diseases, 10th revision (ICD-10) [19] as a source of expression for a problem list can lead to multiple issues. A classification is a partition of reality in a finite set of categories, resulting in a phenomenon called residual aggregation or residual category. For example, *Other specified immunodeficiencies*; *Disorder of pancreatic internal secretion, unspecified*; or even *Fracture of unspecified phalanx of other finger* exist in ICD-10 to cover all concepts that do not fall in another category [20]. This type of term is not suited for a problem list because it does not represent problems that clinicians can reasonably enter.

Another challenge in using a classification as a source is based on its organization, tightly connected to the *intention*, which supported its development. For example, ICD-10 aims to properly express causes of deaths and morbidity, the International Classification of Primary Care, 2nd edition (ICPC-2) [21] focuses on primary care problems, and the Logical Observation Identifiers Names & Codes [22] covers observations and laboratories. Thus, each of these have a specific structure and a dedicated organization of their hierarchy to answer the requirements of their use.

The problem list should be able to represent any of those *intentions*, regardless of their future interpretations according to specific classificatory intentions, and without restricting elements to only one classification nor requiring clinicians to know the organization of all of them. A hierarchy such as the ICD-10 results in choices that will favor some dimensions over others. As an example, there is no infectious disease chapter in ICD-10, which seriously complicates the identification of infectious diseases. As a consequence, our approach focuses on using real-world clinicians’ expressions as the primary source and then manually adding as many *semantically meaningful* dimensions as needed.

Maintenance and updating of problem lists is also challenging. For example, during the current pandemic, it was suddenly required to add several new entries to express the specific spectrum of COVID-19. Such rapid adaptations of the list must be rapidly implemented and should not depend on the update cycle of an international classification.

Using an efficient problem list requires considerable background information. For example, the same problem can be addressed several times. This is sometimes appropriate, such as repeated fractures, and sometimes inappropriate, such as repeating at each encounter that the patient has hypertension. Describing the semantics properly facilitates and speeds up the work of clinicians [23,24]. Semantic dimensions should support recognition and reconciliation algorithms and different views of the list, by specialty, organ, and severity, to name a few [25,26] or to support graph-based, symbolic, machine learning, or clustering algorithms to group concepts along a navigation that answers the needs of clinicians, case managers, researchers, etc [27].

### Implementation and Adoption

Although the advantages of a well-maintained problem list are clear, numerous issues have been raised in the way it should be implemented. Engaging users to document a list of problems for their patients in a complete and efficient manner is a challenge. Clinicians in hospitals are under constant pressure, and the effort to pivot from a free-text problem list to a dedicated EHR module can be important. Factors such as gap reporting, problem-oriented charting, or links to billing codes have shown some positive impact on the completeness of the list documented by clinicians [23,28]. In addition, training and education seem to be key factors in adoption [29-31]. In 2016, Simons et al [32] proposed a list of determinants for the successful implementation of a problem-oriented medical record. It includes the completeness, interoperability, usability, and training of staff.

In this paper, we aim to address the challenges of building and implementing a shared, multipurpose common problem list at Geneva University Hospitals (HUG) using an approach based on the clinician’s language and semantic dimension encoding. The driving concepts of this work are that the content of the list should be created with the care professionals to match their needs and that the list should be mapped to terminologies to (1) improve adoption, with metadata for completers and (2) for secondary use of data [4,33]. After the description of the building, implementation, and iterative improvements of the list, an analysis of its use over 4 years is presented.

## Methods

### Approach

This approach focused on 2 goals. First, it allows clinicians to express themselves freely with a list representing the language used every day in clinical interactions and working with a free-text completer rather than a constrained closed list. Second, it allows the use of the list for multiple purposes in the hospital, other than supporting the care activities of the clinicians. The latter is performed by a back-office multidimensional extension of metadata of free-text expressions.

### Common List Creation

To represent the language of the clinicians, the starting point is sentences expressing problems written by clinicians. The initial list was created based on 2 sets of documents extracted from the HUG’s data warehouse, one from the internal medicine department and the other from the surgery department. Each set was composed of 10,000 admission letters and 10,000 discharge summaries for a total of 40,000 documents. Every natural language sentence in these documents was extracted using automatic tools without further processing. Those sentences were then manually selected if they represented a potential candidate, curated for typos and grammatical normalization such as plural or uppercase reserved for proper names. The abbreviations have been expanded but kept. Rules applied to build this list were inclusive, covering problems of any type, including but not limited to medical, surgical, socioeconomic, psychologic, logistic, etc. Synonymy is allowed, so that multiple expressions expressing the same problem are present, such as *generalized pain* and *pain everywhere* but connected as synonyms. Every granularity is allowed as long as the expression is used by clinicians. The only strict rule is that an expression must be syntactically and morphologically unique.

The list of expressions is improved based on 2 axes: vertical (expressions) and horizontal (dimensions). Extensions of the list require deployment in a specific clinical context, for example, neurosurgery. In this case, discussion with clinicians and analysis of their clinical documents allows us to build a set of specific expressions for that context, which are added to the common list before the deployment. Adjustments of the list are also iteratively made based on use, aided by the fact that the problem list management module is based on a syntactic completer allowing clinicians to enter free text and then select an expression if appropriate, or keep the original free text. The modifications of the list, expressions, and activity state of expressions are fully historicized based on use. Deletions are usually forbidden, which happened only once after a one-year evaluation of the impact of deleting entries: ensuring they had never been used and the impact of their absence on tools such as completers, parsers and colocations, word embedding, etc.

A monthly use analysis with all expressions chosen, by whom, in which context and the potential free text added are used to improve the list.

### Semantic Dimensions

#### Overview

We considered a semantic dimension as any metadata added to the list of expressions to improve its use for a specific purpose. This purpose can be the completer functionalities, for example, for ambiguous abbreviations (in French, *TV* can mean *tachycardie ventriculaire* or *toucher vaginal*), or when the expression is gender specific, such as all expressions relating to *prostata.* Some dimensions are related to national classifications, such as the Swiss Classification for Surgical Interventions (CHOP) [34], or international classifications, such as ICPC-2 or ICD-10, including their various versions (several releases of ICD-10, for example). Finally, some are internal to the organization, such as a specific identification for surgery requiring a surgical theater, used for logistics and resource management at HUG. Expressions can have no or several entries in any specific dimension.

Encoding was performed by domain experts. For example, the ICD-10 and CHOP classifications have been made by a coding expert of the billing division of HUG, SNOMED CT encoding by a physician, ICPC-2 encoding by an outpatient physician, etc. Several dimensions, such as chronic or acute, gender specificity, and syntactical dimensions, have been conducted by medical students.

The dimensions described here are not exhaustive but representative. The coding of the dimensions is a complex activity, mostly toward maintaining global coherence. In this work, the strategy is to have a clear definition of a dimension and aim to reach the best quality of representation of that dimension, regardless of the others except the expression itself. The objective is that a specific expression that can be represented in that dimension must be represented with the highest precision possible for that dimension, respecting only the rules specific to it. This strategy has several advantages. It allows to keep the intention of the dimension to be coded at best and allows the encoding work to be distributed among several actors, domain experts, or students, according to their competences specific to that dimension and their understanding of the expression. Finally, a specific expression can be understood differently and with a different granularity, according to the perspective of the dimension used, or seen as the sum of some or all dimensions.

#### General Classifications

##### International Classifications

*ICD-10* is the basis for the billing of inpatient stays in Switzerland. Once every 2 years, the Swiss Confederation publishes its own version of the ICD-10 classification, which is a translation of the ICD-10, German Modification (ICD-10 GM), which is a slightly modified version of the ICD-10 released by the World Health Organization (WHO) [35]. Every expression in the list was first encoded with the ICD-10 WHO version to evaluate gaps in the list and perform subset definitions for specific use cases. Second, the list was encoded using the Swiss ICD-10 GM version. The encoding was performed using the official coding rulebook for hospital stays in Switzerland [36]. This dimension has been added in the aim of performing automatic coding of inpatient stays for billing, prediction tools for problems versus diagnosis, or support of pathways. Several versions of the ICD-10 are encoded, according to years, or to the source, including the WHO’s original ICD-10.

*ICPC-2* is a classification used to encode general practice clinical activities and primary care. It belongs to the WHO family of international classification [21]. This classification was chosen by the clinicians from the outpatient clinics for its ability to classify problems in simple categories relevant for care, such as symptoms, diagnosis, screening, or procedures. In addition to the activities of outpatient clinics, including research, this classification is used to generate alerts when adding multiple problems with the same ICPC-2 encoding.

The *Systematized Nomenclature of Medicine Clinical Terms (SNOMED CT)* is a term with more than 340,000 concepts and 1 million relationships [7,37]. It is described as the most comprehensive clinical health care terminology in the world and has become central to semantic interoperability. It has been chosen as the United States standard for encoding diagnoses and problem lists [38]. SNOMED CT includes powerful features such as the combination of concepts (postcoordination) or the expression constraint language, which can be used to perform complex queries on SNOMED CT encoded data. SNOMED CT is one of the pillars for the semantically driven activities for data science at HUG and allows the connection of many different aspects of the EHR, such as problem lists, formularies, and other structured data. It allows complex queries, such as every problem related to an organ, or including an inflammatory process. Owing to the size and complexity of the terminology, encoding several expressions in SNOMED CT requires a significant amount of time and experience. The encoding of the expressions uses only single or multiple precoordinated elements, a step toward fully postcoordinated expressions.

##### National Classifications

CHOP [34] is used to encode and bill surgical interventions. Goals similar to those of the ICD-10 GM were added. Every expression in the list that can be mapped to a CHOP code was mapped and updated annually when the new version was released.

#### Internal Classifications

Several internal classifications are used in specific contexts, which are illustrated hereafter.

##### Department or Specialty-Specific Lists

Adult and pediatric emergency departments use specific problem lists, which were included in the process. Most of the time, these lists were derived from the ICD-10. Appropriate dimensions were added, including specialty preferences. The adaptations were systematically validated by specialty experts. The same process has been applied in several specialties, such as oncology and neurosurgery.

##### Clinical Decision Support

Some expressions and dimensions have been added specifically to support computerized provider order entry, exemplified with *antibiotic prescription support* to improve choice of antibiotics, monitor, and lower antibiotic resistance*.* The expressions related to that list were added, properly encoded, and their belonging to problems related to antibiotic prescription added in a new dimension, so that it could be used in several modules of the EHR.

##### Surgical Intervention List

One key development enabled the use of the list as a unique source of expressions for surgical intervention planning and documentation. When an intervention is planned in the hospital, it triggers a chain of events that will lead to the intervention. The operating room must be booked, staff must be appointed, specific devices and materials must be ordered, etc. Historically, this process was separated into silos, medical, paramedical, or logistic with separate lists. The list of surgical interventions used for operating room planning was manually integrated into the common list as a new dimension. This integration was made by specialties and is still ongoing. It allowed the common list to become a single source of expressions for surgical intervention planning.

##### Nutrition and Dietetic Diagnoses List

The most recent development of the list focused on the diagnoses used by the dieticians and nutritionists, which was a list of expressions extracted from the Terminologie Internationale de Diététique et de Nutrition [39]. These expressions were curated and integrated as a new dimension, making the common list the single source of expressions for the nutritionist and dieticians of the hospital.

#### Other Dimensions

Other specific dimensions are useful for numerous purposes. The gender specificity dimension defines whether an expression is gender specific, such as *vasectomy*. The intervention dimension defines an act performed and differentiates it from interventions requiring surgery theater. Multiple other dimensions are used for numerous purposes, such as possible abbreviations of the expression, preferred terms, chronic or acute, etc.

#### Language

The expressions being in French, an English translation was prepared, and keywords of the expressions were added in both French and English.

## Results

### Evolution of the List

The list of problems presented in this work had to *compete* with 17 specific, specialty vertical, local problem lists and was proposed as an additional choice for clinicians. They could freely choose between their *usual* lists and the new one. This competitive approach was a strong incentive to stick to the needs of clinicians and become their *preferred* list. Within the first year, the new list became the most used in most cases, and the legacy lists were then removed. The 2 first years required frequent adjustments, but with a slowing down frequency up to the current situation, which is on specific demand, such as COVID-19, or monthly. Table 1 summarizes the major releases.

**Table 1:** Major releases, corpus size, and comments.

Date of release	Active problems, n	Modifications
January 2017	45,946	First production deployment
September 2017	45,458	Partial integration of expression for surgery planning Corrections of expressions
January 2018	51,255	5867 expressions created from legacy list use and free-text entries
February 2018	50,822	Integration of expressions for antibiotics prescription and monitoring project Corrections of expressions
May 2018	52,040	1091 expressions created from legacy list use and free-text entries
November 2018	52,211	Integration of the list for adult emergency ward Abbreviations system integration
August 2019	51,824	310 expressions created on demand from users
January 2020	52,956	Integration of expressions for surgery planning Integration of a list of diagnoses used by dieticians and nutritionists Integration of the list for pediatric emergency ward
April 2020	52,958	Emergency adding of 2 expressions for SARS-CoV-2 cases
August 2020	20,120	Inactivation of 32,840 never used expressions Preferred term system integration

In January 2017, the list was deployed in the geriatric and general pediatric division of the HUG, as well as part of the rehabilitation medicine division and ambulatory primary care division. The list was then progressively deployed in new divisions. Table 2 summarizes these deployments and Table 3 exposes some descriptive statistics of the current list.

**Table 2:** Deployment of the list in new divisions by date.

Date	Division
April 2017	Neurosurgery
May 2017	Neurology
May 2017	Visceral surgery
November 2017	Psychiatry (adult and pediatric)
November 2018	Rehabilitation
September 2019	Adult emergency
September 2020	Internal medicine
September 2020	Oncology
September 2020	Cardiology

**Table 3:** Some descriptive statistics of the list.

Type of expression or encoding	Expressions (N=20,120), n (%)
Active expressions	20,120 (100)
Abbreviations	2127 (10.57)
ICPC-2^a^ encoding	20,120 (100)
ICD-10^b^ WHO^c^ 2008 encoding	11,860 (58.95)
ICD-10 GM^d^ 2018 encoding	18,481 (91.85)
CHOP^e^ 2019 encoding	1223 (6.08)
SNOMED CT^f^ encoding	9222 (45.83)
Gender specificity encoding	805 (4)
Acute or chronic specificity encoding	8013 (39.83)
Intervention encoding	1855 (9.22)
Surgery planning	985 (4.89)
Antibiotic decision support	553 (2.75)
Adult emergency ward	1108 (5.51)
Pediatric emergency ward	939 (4.67)
Nutrition and dietetics	139 (0.69)

^a^ICPC-2: International Classification of Primary Care, 2nd edition.

^b^ICD-10: International Classification of Diseases, 10th revision.

^c^WHO: World Health Organization.

^d^ICD-10 GM: International Classification of Diseases, 10th revision, German Modification.

^e^CHOP: Swiss Classification for Surgical Interventions.

^f^SNOMED CT: Systematized Nomenclature of Medicine Clinical Terms.

For us, an important success indicator is that currently, 3 major divisions, internal medicine, geriatrics, and rehabilitation, decided to remove free-text entry possibility, judging that the common list was sufficiently complete for their use.

**Figure 1:**
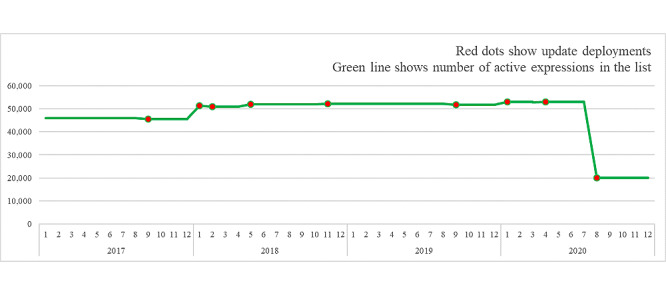
Evolution of the number of active expressions in the common list.

In 4 years, 7270 expressions were added from legacy lists, free-text, or users’ requests. After 3 years of use, all 32,840 expressions that were never used or linked to any specific project were inactivated from the source and deleted for production. The current version of the list contains 20,120 active expressions. The evolution of the number of expressions in the list is shown in Figure 1.

### Use of the List

After 4 years of use, all problems created were extracted from HUG’s data warehouse, representing 1,146,135 problem creations. Among them, 59.69% (684,102/1,146,135) were chosen from the common list, 14.83% (169,970/1,146,135) from legacy lists, and 25.48% (292,063/1,146,135) entered as free-text entries. Over the legacy list problems, 63.01% (107,095/169,970) were created during the first year. In December 2017, the month with the largest proportion of free-text entries, 37.47% (8321/22,206) of the problems were created using this method. In December 2020, the last month of the observation period, 18.38% (4547/24,738) of the problems were created using free text and 80.18% (19,836/24,738) using the list.

**Figure 2:**
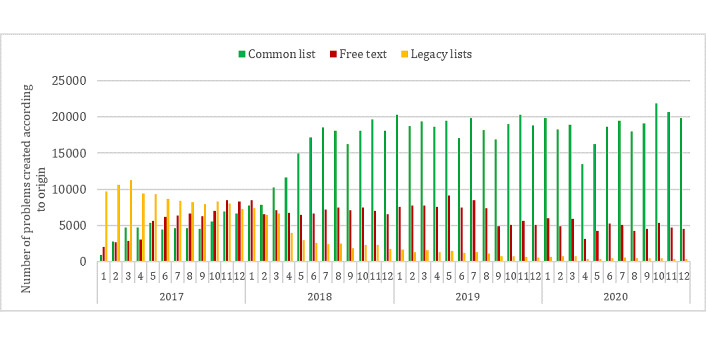
Number of problems created by month according to their origin.

From the common list, 15,232 distinct expressions were used at least once. Figure 2 shows the absolute number of problems created by the month and their origin. Legacy lists combine all problems arising from the 17 legacy lists in production in the HUG at the time of the first deployment and are progressively abandoned.

**Figure 3:**
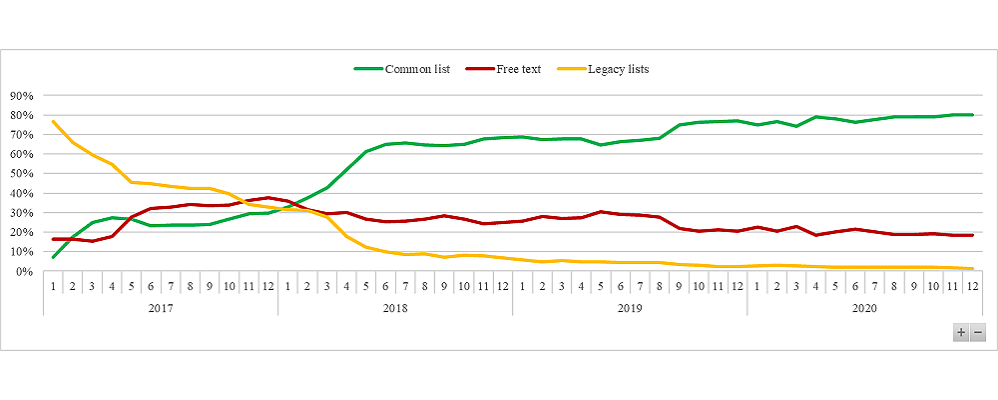
Proportion of problems created by month according to their origin.

Figure 3 displays the proportion of problems chosen in the common list versus legacy lists and free-text entries.

The 20 most frequently used expressions in the list are listed in Table 4. Free-text entries and expressions from the legacy lists were not included.

**Table 4:** The 20 most frequently used expressions of the common list over 4 years.

Expression	English translation	Uses, n
Hypertension artérielle	Arterial hypertension	16,974
Insuffisance rénale aiguë	Acute renal failure	6391
Hypercholestérolémie	Hypercholesterolemia	5219
Accouchement normal d'un nouveau-né vivant par voie basse	Normal vaginal delivery of a liveborn	5045
Appendicectomie	Appendicectomy	4550
Hypertension artérielle traitée	Treated arterial hypertension	4230
Décompensation cardiaque	Cardiac decompensation	4159
Douleur thoracique	Thoracic pain	3707
Hypokaliémie	Hypokalemia	3363
Troubles cognitifs	Cognitive disorder	3323
Hyponatrémie	Hyponatremia	3212
Infection à SARS-CoV-2 (COVID19)	SARS-CoV-2 (COVID-19) infection	3118
Fibrillation auriculaire	Atrial fibrillation	3055
Diabète type 2	Type 2 diabetes	3051
Insuffisance rénale chronique	Chronic renal failure	3002
Malnutrition protéino-énergétique grave	Serious protein-energy malnutrition	2898
Dyslipidémie	Dyslipidemia	2844
Obésité	Obesity	2833
Asthme	Asthma	2756
Douleur abdominale	Abdominal pain	2749

Finally, the list was exploited for various research activities, training machine learning models using various mappings, predicting billing codes of a stay using the ICD-10 encoding, or for workload predictions during the multiple waves of the pandemic. This work has not been discussed in this paper.

## Discussion

### Principal Findings

After 4 years of deployment and iterative improvements, a list of 20,120 active expressions mapped to more than 14 semantic dimensions was deployed in most major divisions of HUG and used to create 684,102 new problems. Specific dimensions allowed the list to be used for various purposes, such as surgical planification, decision support or nutrition, and dietetic diagnosis.

Manually building a problem list is a time-consuming task and starting from the clinician language as a source of expressions is a double-edged sword. It aims to improve information precision to support clinicians in finding the most appropriate expressions that best represent the conditions of patients. Cost tends to increase noise when proposing numerous expressions with small variation, syntactically, semantically, or both, depending on the completer used. This effect can be mitigated using the features of the dimensions, either syntactically, such as abbreviations and common variants, or semantically, by using the aggregation properties of classificatory dimensions. This allows us to search for the entirety of the list while reducing the number of possibilities proposed to the most pertinent set. These tools were not discussed in this work. Finally, statistics on the use of the list are important to improve it, such as progressively filtering out never used expressions or improving granularity in existing ones that are extended by free texts, for example.

We noticed that problems appearing frequently in practice tend to have multiple variations, with various levels of granularity or additional information, naturally improving the expressiveness of the list and the ease for clinicians to find the most appropriate element. On the other hand, rare problems tend to have fewer representations, if any in the list thus reinforcing the need to keep free-text entries.

The many dimensions that have been encoded allow the comparison of the list of expressions and their coverage for the respective coverage of classifications. For example, taking ICPC-2 and ICD-10, the immediate observation is that the list contains elements that can be expressed in both classifications, but in many more lexical variants. On the other hand, many classifications are not found in the list, for many of them not codable elements or unmet conditions in our setting. As a result, the list covers more than any of the classifications separately but only meaningful expressions. Moreover, it frees care professionals from the task of knowing multiple classifications and their structures. This reduces the compression of information while maintaining strong interoperable capabilities through semantic dimensions.

Semantic dimensions are a major addition of this approach. They bridge the need for various representations of a concept as expressed by clinicians with the need for semantic interoperability. By encoding each expression into all relevant dimensions, it was possible to reuse the created problems for other goals, for example, by extracting subsets related to a specific disease through ICD-10 encoding, all patients that undergo a specific procedure using the CHOP encoding or more complex queries such as all problems that include an inflammation process through the SNOMED CT encoding. However, the maintenance costs of these dimensions are important. The more dimensions there are, the more work it requires to add a new expression, as it must be encoded in possibly all of them. Moreover, classification updates (such as a new version of the ICD-10) sometimes require a full reading and update of the encoding.

The semantic dimensions linked to intrahospital use cases allowed the list to be used for multiple projects. Specific subsets for divisions, such as emergency wards, were beneficial for convincing users to start using the common list. The surgical planning addition promoted the list as a central source of expressions and concepts outside of the care domain. The role of the list as a central source of expressions for patients’ problems is shown by the number of projects that included the addition of a dimension to the list. In a virtuous circle, the more the list was known, the more demands were made to adapt it to new needs.

As every project of this type, the final challenge is to convince users to use the module and teach them how to do so correctly. This has been heavily pushed in this work by the Medical and Quality Directorate, the team designing the problem list module in the HUG. Teaching both in person and through videos helped disseminate the use of the module in divisions that historically did not use it.

During the first year of deployment, the module was introduced and promoted in 4 new divisions of the hospital. This increases the number of users and the number of problems created. Those new users with no experience of the problem module are arguably the reason for the initial augmentation in the proportion of free-text entries seen in Figure 3. The diminution in problems created from the legacy list is to account for the progressive removal of those lists from the module. After this initial period, the proportion of free text diminishes progressively from 37.47% (8321/22,206) in December 2017 to 18.38% (4547/24,738) in December 2020, the lowest percentage in the full period. It is interesting to note that this period of 1 year also corresponds to the time it took for the common list to become the most used method for creating problems.

This reduction in the proportion of free-text entries shows that the common list corresponds to the needs of care professionals and that its adoption is progressing. Although it is not possible to determine the proportion of this evolution because of the content of the list, the functionalities of the problem module, or the dissemination effort, it seems likely that it is a combination of the three, and that only a transversal approach could succeed in this transition.

The situation before the deployment of the common list seemed preferable because the proportion of free-text entries was low and the use of legacy list was well-established. However, the final situation is arguably better for several reasons. First, the legacy lists lacked proper semantic interoperability. They were manually modified versions of existing classifications, with the limitations described before and the added complexity of manual, unverified modifications. They were not harmonized, and it was not possible to group or analyze problems from multiple lists without manual reading of the expressions. This prevented those lists from being used for other purposes, as the common list allows.

The apparent decrease in the number of problems created in April and May 2020 is explained by the COVID-19 pandemic. Indeed, the HUG stopped their elective activity and shifted to treating only patients with COVID-19, which reduced the number of patients with various problems and reduced the overall number of problems created.

### Sustainability

Sustainability is an important aspect of large-scale projects, such as the creation of a common multipurpose problem list. For this specific issue, a common list presents interesting properties. As explained before, it can be extended vertically in 2 axes by adding new expressions and horizontally by adding new dimensions. This allows the list to quickly integrate new expressions, such as during the COVID-19 pandemic, or new dimensions such as dietetics and nutrition diagnoses. However, the amount of work required for vertical or horizontal extensions is not the same. A new expression can be encoded in all dimensions in a matter of minutes; however, in the worst case, the addition of a dimension requires going through every expression. Although, as the list has been kept to a manageable size by focusing only on expressions used in practice, this work can be performed with reasonable resources. Therefore, this list presents good flexibility and sustainability.

### Reproducibility

The approach taken in this study was focused on the language of the clinicians. Therefore, the list of expressions is highly dependent on the clinicians, their language, their cultural background, and the population they cover. Therefore, the list itself will always be the most useful in the hospital where it has been created. However, the approach proposed to create the list is reproducible in any hospital wanting to create a problem list and for other use cases where a controlled vocabulary can be used but does not fit the language used in practice by caregivers.

### Lessons Learned

This work allowed us to draw significant learning for the building and implementation of a problem list. These are listed in Textbox 1.

**Key learnings.**

**Key learnings**

- Existing controlled vocabularies are too narrow or subject oriented to be used natively as problem lists.
- It is possible to build a problem list starting from the clinician’s language to better match their needs.
- It is possible to reduce the expressivity needed for a problem list to a meaningful set of expressions used in practice.
- On purpose semantic dimension encoding allows secondary use of data.
- Internally building a list of expressions allows flexibility and quick adjustments when needed.

### Limitations

Although the data and analysis included in this work were carefully carried out, some limitations are worth noting. First, the evaluation data were analyzed as a source of problems created. However, this does not translate to the complexity of the deployment of the list in the hospital. Indeed, the problem module is deployed in the EHR globally, but some divisions use it, while others do not. Inside these divisions, some teams of residents are more used to the module than others. Additional data should be gathered to track the dissemination effort, the training provided, to understand when the module was adopted in which division and by whom.

Finally, the proportion of common list, free text, and legacy list problems is only a proxy for user preferences. It does not account for other elements, such as division-specific guidelines or orally transmitted habits. To credit the progression of the common list to its quality is a conclusion that should be confirmed by a closer evaluation, in partnership with the users.

### Conclusions

Overall, there is still room for improvement when building and implementing a problem list in the production environment of care. Most of the existing efforts use terms from existing terminology rather than focusing on the language used by clinicians. The perfect problem list that contains what care professionals want and can be used for every other use-case is yet to be created.

The proposed approach breaks with common approaches for the building of problem lists by directly addressing the gap between existing controlled vocabularies and real clinicians’ language when expressing a patient’s problem. Second, it brings new perspectives for secondary use by encoding the expressions in various semantic dimensions, allowing specific uses of the list in the hospital and beyond.

By applying this approach, more than 50,000 expressions were manually curated into a common problem list integrated in the EHR. Through iterative updates, the list was enriched and refined to 20,120 active expressions matching users’ needs. More than 14 semantic dimensions were added to the list, including 5 major classifications and multiple dimensions internal to the hospital, such as division-specific adaptations, surgical planning, antibiotic prescription support, nutrition, and dietetic diagnoses. These additions pushed the adoption of the common list as a central, harmonized source of expression in the hospital. The recent decision of 3 major divisions of the hospital to remove the option to make free-text entries shows that the list corresponds to the needs of the users.

Manually creating and updating a set of expressions directly extracted from clinical documents has succeeded in HUG to engage users in transitioning from legacy systems to a new module including the common list. The overall number of problems created is increasing, while the problems entered as free text are decreasing.

The manual work required to build and maintain the list is substantial in the 3 domains, maintenance of the expressions, development of the problem module, and dissemination of its use. However, this approach provides a solution for keeping data interoperable while not constraining the user and allowing multiple use cases.

Moreover, with the large adoption of the list in the HUG, new perspectives open and new types of projects are possible. Ongoing developments include oncologic diagnoses with the addition of a dimension mapping expression to the third edition of the International Classification of Disease for Oncology, extension of the surgical planning dimension, or creation of the 2021 version of the ICD-10 GM and CHOP dimensions. The addition of dimensions for new international classifications, such as the eleventh revision of the ICD, are also evaluated. However, the improvement of the SNOMED CT dimension is currently prioritized over these additions, owing to the quantity of information expressible in SNOMED CT, the multiple mappings existing between SNOMED CT and other controlled vocabularies, and the national recommendation of this terminology for interoperability in Switzerland.

In addition, the common list allows new research projects in the medical domain, such as analysis of the problems documented for patients with COVID-19 or focusing on the language, such as the study of the search terms entered by clinicians compared with the problem selected in the list.

An evaluation of the impact of the list on the workload of clinicians and on the secondary uses of the produced data should be made to further validate the approach.

## Abbreviations

CHOP: Swiss Classification for Surgical Interventions
EHR: electronic health record
HUG: Geneva University Hospitals
ICD-10: International Classification of Diseases, 10th revision
ICD-10 GM: International Classification of Diseases, 10th revision, German Modification
ICPC-2: International Classification of Primary Care, 2nd edition
SNOMED CT: Systematized Nomenclature of Medicine Clinical Terms
WHO: World Health Organization
