# Addendum: Building a Shared, Scalable, and Sustainable Source for the Problem-Oriented Medical Record: Developmental Study

**DOI:** 10.2196/41257

**Published:** 2022-08-09

**Authors:** Christophe Gaudet-Blavignac, Andrea Rudaz, Christian Lovis

**Affiliations:** 1 Division of Medical Information Sciences Geneva University Hospitals Geneva Switzerland; 2 Department of Radiology and Medical Informatics University of Geneva Geneva Switzerland; 3 Medical and Quality Directorate Geneva University Hospitals Geneva Switzerland

In “Building a Shared, Scalable, and Sustainable Source for the Problem-Oriented Medical Record: Developmental Study” (JMIR Med Inform 2021;9(10):e29174), the authors made two updates.

After the publication of the original article, the Geneva University Hospitals Common Problem List was released to the public under the Creative Commons CC BY-SA 4.0 license. It was important to the authors that the readers of the article knew that the list was available for reuse.

1. Accordingly, the following sentence was added at the end of the *Acknowledgments* section:

The common problem list is available under the Creative Commons CC BY-SA 4.0 license on Yareta, the digital solution of the University of Geneva for archiving and preserving research data [40].

2. Full citation of this new reference [40] has been added to the article's *References* section.

The correction will appear in the online version of the paper on the JMIR Publications website on August 9, 2022, together with the publication of this correction notice. Because this was made after submission to PubMed, PubMed Central, and other full-text repositories, the corrected article has also been resubmitted to those repositories.
